# Smartphone-Based Answering to School Subject Questions Alters Gait in Young Digital Natives

**DOI:** 10.3389/fpubh.2020.00187

**Published:** 2020-06-09

**Authors:** Carlotta Caramia, Carmen D'Anna, Simone Ranaldi, Maurizio Schmid, Silvia Conforto

**Affiliations:** Engineering Department, Roma Tre University, Rome, Italy

**Keywords:** smartphone use, texting, adolescents, gait parameters, risk of injury

## Abstract

Smartphone texting while walking is a very common activity among people of different ages, with the so-called “digital natives” being the category most used to interacting with an electronic device during daily activities, mostly for texting purposes. Previous studies have shown how the concurrency of a smartphone-related task and walking can result in a worsening of stability and an increased risk of injuries for adults; an investigation of whether this effect can be identified also in people of a younger age can improve our understanding of the risks associated with this common activity. In this study, we recruited 29 young adolescents (12 ± 1 years) to test whether walking with a smartphone increases fall and injuries risk, and to quantify this effect. To do so, participants were asked to walk along a walkway, with and without the concurrent writing task on a smartphone; several different parameters linked to stability and risk of fall measures were then calculated from an inertial measurement unit and compared between conditions. Smartphone use determined a reduction of spatio-temporal parameters, including step length (from 0.64 ± 0.08 to 0.55 ± 0.06 m) and gait speed (1.23 ± 0.16 to 0.90 ± 0.16 m/s), and a general worsening of selected indicators of gait stability. This was found to be mostly independent from experience or frequency of use, suggesting that the presence of smartphone activities while walking may determine an increased risk of injury or falls also for a population that grew up being used to this concurrency.

## Introduction

In the modern world, the use of a smartphone has become a main characteristic of people's lives (1). In particular, children and teens have grown up with an easy and early access to mobile phones; for this reason, they are part of the group of the so-called “digital natives” (2). For young adolescents, the smartphone is an important tool for communication, education, and entertainment purposes (3); moreover, at that age, the web-based social networks built around a mobile app represent the main connection between peers (4). As a consequence, young adolescents typically spend more than 3 h a day interacting with their smartphone (5), and this practice leads them to get familiar with using it while doing a variety of physical tasks (6). Considering this, the use of the smartphone as a concurrent task during activities of daily living is quite common among young adolescents, regardless of the risk that it can represent; for these subjects, texting is the most frequent activity during walking, given its central importance in social network applications (4).

For young and older adults, it has been demonstrated that the use of a smartphone during everyday walking is increasingly resulting in injuries for pedestrians at all ages (5), in a way similar to the effect of texting and internet navigation while driving (5). While being generally considered as automatic, walking requires attention resources (7) and it is governed by a number of higher cognitive processes (8). The main agreed source of risk associated with smartphone use while walking is identified in its distracting power (9); it has however been demonstrated that most gait parameters linked to stability and to fall risk are also altered in controlled laboratory settings (10), where distractions do not represent the main source of injury risk. Thus, risk may increase also as a consequence of biomechanical alterations.

From a biomechanical point of view, the presence of a secondary task determines posture alterations (11) and a higher risk of fall when walking (12); this is commonly associated with variations in gait patterns, such as specific spatio-temporal and stability parameters of gait (13, 14). Moreover, gait alterations were found to be greater in children and adolescents (15, 16) than in adults (17), suggesting the idea that this effect is the result of different concurring phenomena that cannot be generalized across different age groups.

Among secondary tasks, smartphone use while walking has been increasingly studied (18), given its importance in the everyday life of people of all ages. However, no studies have tried to quantify this effect in a younger population of digital natives yet. In this paper, we recruited a population of digital native young adolescents (11–13 years old) in order to check whether smartphone use during gait has a significant effect on the aforementioned parameters and to quantify these variations. The question that we want to answer with this study is whether, given the rather high experience in texting while walking for this age group, the effects that the concurrent task plays on gait performance are negligible in terms of injury risks.

## Materials and Methods

### Participants

Twenty-nine young adolescents (15 girls and 14 boys, age 12 ± 0.5 years, height 1.56 ± 0.08 m) were recruited from a local secondary school: none of them had special educational needs or certified disabilities. Participants and parents were informed about the procedure, and informed permission of parents was obtained before performing the experiments. The protocol was designed in accordance with the Declaration of Helsinki and approved by the local ethics committee (Applied Electronics section of the Engineering Department).

### Procedure

Participants were asked to walk along a 12-m long straight path under two different conditions:

- Baseline: walking at a self-selected speed with no additional concurrent task. No specific instructions added.
- Smartphone: walking while texting messages to the experimenter using an instant messaging app on the smartphone. The concurrent activity involved answering questions sent by the experimenter and taken randomly from a specified list.

The list of questions was defined by the teachers from the Mathematics and English language syllabi of the class the participants were attending. The teachers provided math questions (e.g., “What is the area of a trapeze?,” “What is the area of a rectangle?”) and translation exercises (e.g., “Translate the following verbs in the English language”). The participants were informed about the fact that they would be asked questions regarding the subjects while walking, but no specific information on the questions was given in advance to them. Each participant received the same number of questions.

Prior to the experiment, participants were asked two questions regarding smartphone expertise and frequency of use: (A) How long have you been using a smartphone? (B) How many hours a day do you use it? They were then shown the path to follow and instructed on the activity to perform. Specifically, for the Smartphone condition, they started to walk just after receiving and reading the first question. All the participants used the same smartphone, and a brief familiarization period was allowed. The order of the two conditions was randomized. While no explicit indication on how to handle the smartphone was given, during the experiments all the participants used a two-handed grip to text while walking.

### Instrumentation

**Figure 1:**
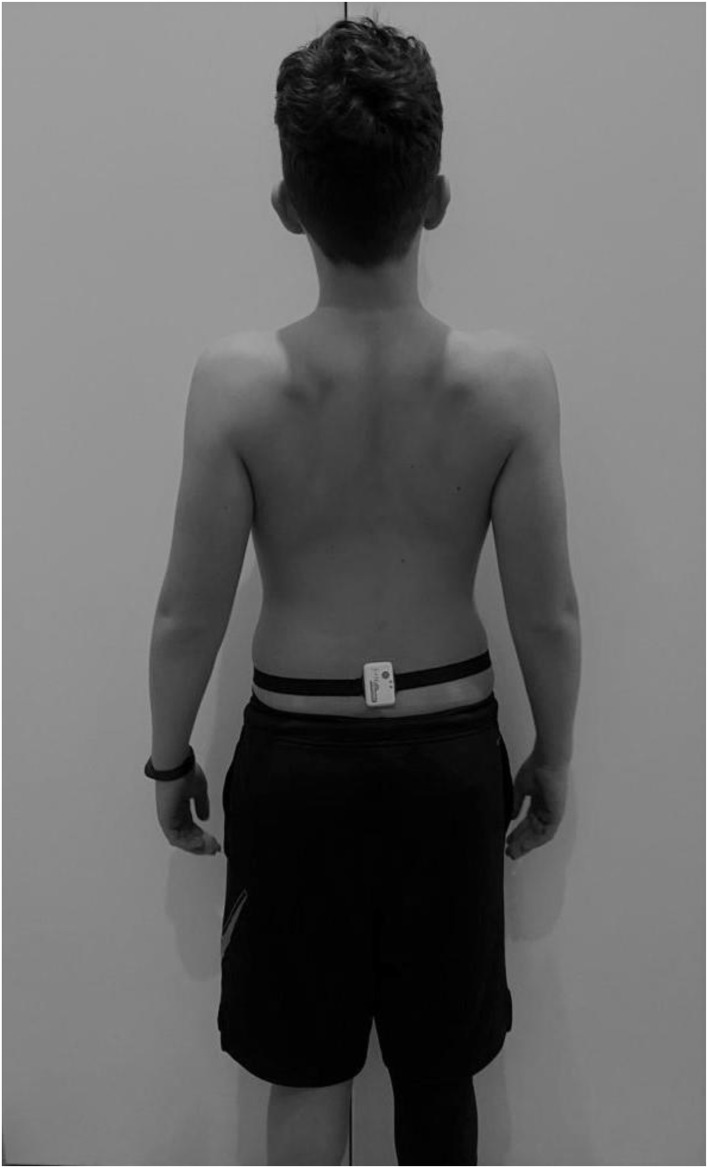
Inertial sensor placement for the experimental procedure.

A single triaxial accelerometer (Shimmer3, Shimmer Sensing, Dublin, Ireland) was placed on the back of the lumbar zone around L3 (19), through an elastic belt (see Figure 1), to acquire linear accelerations along the three main directions (anteroposterior, AP; mediolateral, ML; vertical, VT), in the range ±2 g. Sampling frequency was set at 102.4 samples/s, and data were stored on an on-board SD card. During the experiment, notes were taken to record possible deviations from the defined path or from the activity required to be performed, so as to exclude them from the analysis.

### Data Processing and Parameters Extraction

After realignment with global coordinates, accelerometer data were low-pass filtered with a cut-off frequency of 20 Hz (Butterworth, 4th order), and segmented into gait cycles based on the method proposed by McCamley et al. (20); initiation and termination steps were removed to exclude gait cycles affected by the presence of acceleration and deceleration phases. For each gait cycle, the following gait parameters, arranged into two groups, were extracted.

#### Spatio-Temporal Parameters

- *step length* (m), estimated following the inverted pendulum model (21);
- *step time* (s), the time interval between two successive initial contacts of different feet (22);
- *stride frequency* (Hz), obtained from the power spectra of the acceleration components (23);
- *gait speed* (m/s), the ratio between step length and step time (23).

Overall, decreases in values of these spatio-temporal parameters have been linked to a diminished progression performance, and have been associated with an increased risk of falls in elderly adults (24). Moreover, normalized versions of step length and gait speed with respect to height were also calculated, to exclude any dependence on the results from height. For all spatio-temporal parameters, values were extracted from each gait cycle, and averaged along the whole trial for each condition.

#### Gait Stability Indicators

From the normalized autocorrelation function of the accelerometer data, along the three directions, the following gait stability indicators were calculated:

- *step symmetry*, outlining similarity in walking patterns between left and right steps (25);
- *step regularity*, referring to the similarity between successive left (or right) steps (25);
- *stride regularity* indicating the similarity between successive strides (25).

For the vertical and anteroposterior directions, step symmetry is given by the ratio of the first and second amplitude positive peak at time lags different from zero, while for the mediolateral direction, it is given by the ratio of the first negative amplitude peak at time lag different from zero. Step regularity is represented by the value of the first amplitude positive peak (the second one for stride regularity) for the vertical and anteroposterior components, and by the value of the first negative peak for the mediolateral one (the first positive amplitude peak for the stride regularity).

In general terms, it is expected to have values of symmetry and regularity closer to 1 in normal conditions; values away from 1 may indicate a decrease in gait stability (26, 27).

Then, to use a gait speed-independent measure of walking smoothness, from each component of the acceleration vector the following indicator was calculated:

- *spectral arc length* (SPARC), as a measure of walking smoothness (28).

SPARC quantifies smoothness by computing the negative value of the arc length of the normalized Fourier spectrum of the modulus of the acceleration signal, in the frequency range of the movement. The maximum frequency for SPARC calculation has been defined as the frequency above which the normalized spectrum remains lower than 0.01 (29). A smoother gait pattern results in a higher (i.e., closer to zero) value of SPARC. In normal gait patterns, SPARC results were higher than in the presence of pathologies (29).

For all the gait stability indicators, values were calculated by considering the whole trial for each condition.

#### Smartphone Use Habit Sub-grouping

All participants declared they used the smartphone every day. To assess whether frequency of use or smartphone expertise were factors in possible modifications on gait behavior, the analysis on both spatio-temporal gait parameters and gait stability indicators was done by splitting participants into sub-groups considering reported years of use (question A) and frequency of use (question B). In particular:

- A1 subjects have been using the smartphone for 1–2.5 years
- A2 subjects have been using the smartphone for more than 2.5 years
- B1 subjects (moderate users) regularly use the smartphone up to 2 h/day
- B2 subjects (frequent users) regularly use the smartphone for more than 2 h/day.

### Statistical Analysis

Descriptive statistics included measures of central tendency and dispersion, and it was calculated for each parameter under both conditions. The distribution of data for each parameter was tested for normality by group using Lilliefors test. To check for the presence of an effect during texting, a one-way ANOVA (with condition as factor) was performed on the gait parameters that showed normality. If normality was rejected at the chosen significance level, a Kruskal-Wallis test was used. To study the effect that the years and frequency of smartphone use could have on the gait parameters, two 2-way ANOVA tests were applied considering the condition (baseline/smartphone) and either smartphone expertise (A1 or A2) or frequency of use (B1 or B2) as factors. Tests significance was set at 0.05.

## Results

Mean, standard deviation, and *p*-values for all extracted parameters are shown in Tables 1, 2.

**Table 1:** Descriptive statistics for the spatio-temporal parameters (group mean ± standard deviation), and results of the corresponding statistical analysis.

**Spatio-temporal parameters**	**Baseline**	**Smartphone**	***p*-value**
Step length (m)	0.64 ± 0.08	0.55 ± 0.06	<0.001
Normalized step length	0.41 ± 0.04	0.35 ± 0.04	<0.001
Step time (s)	0.53 ± 0.03	0.61 ± 0.06	<0.001
Stride frequency (Hz)	0.96 ± 0.06	0.83 ± 0.09	<0.001
Gait speed (m/s)	1.23 ± 0.16	0.90 ± 0.16	<0.001
Normalized gait speed (s^−1^)	0.79 ± 0.10	0.58 ± 0.11	<0.001

**Table 2:** Descriptive statistics for the gait stability indicators (group mean ± standard deviation), and results of the corresponding statistical analysis (n.s. for *p*-value > 0.05).

**Gait stability indicators**	**Baseline**	**Smartphone**	***p*-value**
Step symmetry AP	1.00 ± 0.11	1.11 ± 0.11	<0.001
Step symmetry VT	0.99 ± 0.11	1.13 ± 0.27	0.003
Step symmetry ML	−0.91 ± 0.26	−1.07 ± 0.31	0.01
Step regularity AP	0.78 ± 0.09	0.72 ± 0.11	<0.001
Step regularity VT	0.80 ± 0.10	0.67 ± 0.18	<0.001
Step regularity ML	−0.49 ± 0.12	−0.48 ± 0.14	n.s.
Stride regularity AP	0.79 ± 0.09	0.66 ± 0.14	<0.001
Stride regularity VT	0.82 ± 0.11	0.62 ± 0.20	<0.001
Stride regularity ML	0.56 ± 0.14	0.47 ± 0.13	0.01
SPARC AP	−4.22 ± 0.08	−4.29 ± 0.09	0.002
SPARC VT	−4.26 ± 0.12	−4.26 ± 0.07	n.s.
SPARC ML	−4.27 ± 0.07	−4.34 ± 0.07	<0.001

### Spatio-Temporal Parameters

A significant effect driven by the use of the smartphone appeared for all spatio-temporal parameters. In particular, the use of smartphone during walking increased the step time, and decreased step length (and its normalized version), stride frequency, and both versions of gait speed. The numerical results are reported in Table 1.

### Gait Stability Indicators

When using the smartphone, the statistical analysis on symmetry parameters yielded a significant increase of the step symmetry components along the anteroposterior and vertical directions, while a significant decrease appeared for the mediolateral direction. Most gait regularity parameters decreased significantly in the smartphone use condition, with only the step regularity along the mediolateral direction being unaffected. Gait smoothness in the vertical direction was not affected by the presence of the concurrent task, which, in turn, led to significantly lower smoothness in both components of the transverse plane. The corresponding numerical results are reported in Table 2.

### Effect of Smartphone Use Habit on Gait Parameters and Indicators

The questionnaire answers showed that 10 participants have been using a smartphone up to 2.5 years (sub-group A1), while 19 for more than 2.5 years (sub-group A2); 14 individuals reported using the device for up to 2 h/day (sub-group B1), the remaining 15 declared regular use of more than 2 h/day (sub-group B2).

The statistical analysis showed no significant modifications of any spatio-temporal parameter based on either sub-group splitting (for both A and B). A significant modification of some gait stability indicators based on frequency of use (sub-group B) appeared. In particular, step regularity along antero-posterior and vertical direction, and stride regularity along the antero-posterior direction were all significantly higher for frequent users, as compared to moderate users; likewise, SPARC resulted lower for frequent users (see Table 3). No significant effect from years of use appeared. Both spatio-temporal gait parameters and gait stability indicators were dependent from condition in both sub-groups, while no interaction between condition and either frequency or years of use was found.

**Table 3:** Descriptive statistic (group mean ± standard deviation) and *p*-value for the gait stability indicators influenced by condition and frequency of use (n.s. denotes *p*-value > 0.05).

**Gait stability indicators**		**Moderate users**	**Frequent users**	**Main effect (condition)**	**Main effect (frequency of use)**	**Interaction (condition × frequency of use)**
Step Regularity AP	Baseline	0.74 ± 0.09	0.82 ± 0.05	*p* < 0.01	*p* = 0.007	n.s.
	Smartphone	0.67 ± 0.10	0.76 ± 0.09			
Step Regularity VT	Baseline	0.75 ± 0.08	0.85 ± 0.08	*p* < 0.001	*p* = 0.04	n.s.
	Smartphone	0.62 ± 0.19	0.71 ± 0.14			
Stride Regularity AP	Baseline	0.77 ± 0.08	0.80 ± 0.09	*p* < 0.001	*p* = 0.04	n.s.
	Smartphone	0.61 ± 0.15	0.71 ± 0.11			
SPARC AP	Baseline	−4.21 ± 0.08	−4.24 ± 0.08	*p* < 0.001	*p* = 0.008	n.s.
	Smartphone	−4.25 ± 0.09	−4.34 ± 0.06			

## Discussion

This study aimed at determining the influence of smartphone use while walking on a variety of gait parameters recorded on a population sample of young adolescents. Technology is a constant part of their everyday life and their approach to smartphone use started at a young age (30). Despite their familiarity and expertise with the use of such devices, we were able to confirm that smartphone use during walking determined a variation of multiple gait parameters, including measures of gait symmetry, regularity, and smoothness.

### Spatio-Temporal Parameters

When using smartphone while walking, step time increased and step length decreased, which is indicative of a slower walk. This is similar to results obtained in multiple dual-task studies on gait involving children of different age ranges (31–33). In the presence of a concurrent task, young individuals tend to walk slower and with smaller steps, as do adults. In terms of effect size, we could not draw a direct comparison with published research on the elderly (34) and young adults (35), given the specific nature of the additive concurrent task employed in this study; however, the relative reduction we observed on gait speed corresponds to the upper limits of the reported range of reduction in adults (34), thus suggesting that the effect on the studied age group is relevant. Regarding spatio-temporal parameters, we could not exclude that modifications of gait speed and step length may also depend on the altered posture caused by handling the phone, as disentangling purely postural effects from cognitive ones would have needed a “mock” condition where subjects were requested to handle the phone without answering questions. However, the amount of changes caused by maintaining a fixed elbow has been quantified in around 0.03–0.05 m/s (36), thus well below the overall effect observed in this study. These findings suggest that the nature of these modifications is mostly determined by the attention share of the secondary task.

A significant decrease also appeared for the normalized version of step length, thus highlighting that step reduction is independent from height.

We could speculate that such an amount of reduction might be associated with the adolescents prioritizing texting over motor function, and that the significant alteration of all spatio-temporal parameters might be linked to a decrease in attention to the surrounding environment.

### Gait Stability Indicators

All gait stability indicators showed a worsening caused by texting: participants showed a less symmetrical, less regular, and less smooth gait. In particular, gait symmetry in the sagittal plane was detrimentally affected; while we could not exclude a higher involvement of the dominant hand when texting on the smartphone, we were positive of the absence of visible postural trunk asymmetries. In this, we were supported by the observation that all the involved individuals used the smartphone in a 2-handed holding configuration.

The observed decrease of regularity parameters mainly in the sagittal plane is in line with what has been found in a variety of dual-task studies involving adults and the elderly (12, 35), and it has been directly linked to increased task-related motor and cognitive demand, as confirmed by a higher central involvement when texting while walking (37). The hypothesis of a similar involvement also in the observed adolescent sample might explain our findings on regularity parameters.

Variations of smoothness in both components of the transverse plane may be linked to a less adaptive walking pattern, since both SPARC indicators showed a decrease when texting. Even if the effect coming from the presence of the concurrent task on SPARC is rather low, we outline here that this metric has been reported to be found in robust to walking speed variations (28, 29), and it can thus accurately capture differences in smoothness that are not the result of step time variations. The decrease of these measures has been linked to less steady walking patterns and it has been hypothesized as a predictor for fall risk in people with Parkinson's disease (29); changes in smoothness were interpreted as caused by the competition for resources between cognition and gait (38) and for the reduced visual fixation time at the travel path (39).

### Smartphone Use Habit and Implications for Fall and Injury Risks

We observed that being a digital native does not protect from risks identified in older populations when texting while walking, and this is aligned with observed modifications on selected gait parameters in a similar population (40). Despite the different level of familiarity with smartphone use (39), we found that the effect of this concurrent task for digital natives resembles the one reported in the literature on samples of young adults. We also observed that no difference appeared on spatio-temporal parameters between more familiar users and less familiar ones, and this result may confirm the hypothesis of an experience-independent effect of the secondary task on gait. However, we found some elements of difference between the frequent users and the others, i.e., frequent users displayed higher regularity, at the expense of a reduced smoothness. We do not have a clear explanation for these results, but it may be speculated that frequent smartphone users have a tendency to base on rhythmicity when walking, with a reduced emphasis on stability indicators, such as smoothness. We stress here that the reduced statistical power of the analysis, when performed on each sub-group, prevents us from formulating robust interpretations on this.

One of the main factors for observed changes of gait parameters in adults has been hypothesized in the tendency to prioritize texting over walking (41). While we could not directly apply this hypothesis to the observed young adolescent population sample as we did not collect error data on the concurrent task, one possible explanation would be the following: while digital natives may be more used to texting while walking, they may not be efficient enough in governing the concurrency between the activities, according to the reported observation that they do not have a higher ability to multitask than digital new-comers, who are a generation of people that acquired familiarity with a smartphone as adults (2). The ability to multi-task effectively has in fact not been directly linked to the frequency of engagement in multiple tasks simultaneously (2). As a result, young adolescents too may prioritize texting over walking, because they are very proficient in the smartphone use. Since we did not directly collect data on the number of text errors made, we could not verify this hypothesis. At the same time, we could not exclude that another effect may come into play: the agreed overconfidence displayed by young adolescents in a variety of tasks, as compared to adults (42). Being involved in multiple tasks, they may experience high interference on gait, as their ability to execute both functions may be lower than the self-perceived one. It may be interesting to verify if this overconfidence phenomena exacerbates in the presence of elements of disturbance to gait (39). We could not exclude that, in this process, a possible role may be played by the development of motor and cognitive functions being non-complete at this age (43), also in terms of the ability to govern the attentional resources required to control gait (15).

Regarding gait parameters as possible predictors for the risk of injury and fall, it has been shown that most parameters of gait actually change secondary to the main observed change, the decrease of gait speed (44, 45). While we could not exclude that this may be also the case for many observed parameters in our study, the presence of modifications on a substantially velocity-independent parameter of smoothness, i.e., SPARC, comforted us on the validity of the findings. Other measures of smoothness were linked to an increased risk of falls in elderly adults (46). Even if, to our knowledge, a thorough test of the link between SPARC values and the risk of fall is still missing from the literature, the robustness of SPARC with respect to gait speed variations, and the presence of an effect coming from a secondary task on this parameter for the studied population, could call for new studies on this topic.

Walking behavior while using a smartphone is altered in young adolescents. Despite the familiarity of this age group with the everyday use of such devices, the concurrent use of smartphones during gait determines a general worsening of those parameters that are associated with gait performance and stability; as a matter of fact, we observed a general decline of gait speed to values that are lower than 1.1 m/s, a value which is suggested as a threshold of safety in crossing roads for pedestrians (47, 48). Considering this, we can conclude that this kind of concurrent task on walking in this population might lead to biomechanical alterations and decreased stability; in addition, the non-complete motor control development may amplify the effect of a different cognitive load while walking, increasing all the risks associated with smartphone use during daily life.

## Data Availability Statement

The datasets generated for this study are available on request to the corresponding author.

## Ethics Statement

The studies involving human participants were reviewed and approved by Applied Electronics section, Department of Engineering, ID 02/18. Written informed consent to participate in this study was provided by the participants' legal guardian/next of kin.

## Author Contributions

The study was designed by CC, MS, and SC. Recruitment and data collection was run by CC and SR. Data processing and statistical analyses was conducted by CC, CD'A, and SR. The manuscript was written by all authors. CC drafted the Material and Methods and Results sections. MS, SC, CD'A, and SR drafted the Introduction and the Discussion. CD'A led the additional analysis on subgroups and the revision to the original submission. The final version was approved by all authors.

## Conflict of Interest

The authors declare that the research was conducted in the absence of any commercial or financial relationships that could be construed as a potential conflict of interest.
